# Refining Mitochondrial Intron Classification With ERPIN: Identification Based on Conservation of Sequence Plus Secondary Structure Motifs

**DOI:** 10.3389/fmicb.2022.866187

**Published:** 2022-03-18

**Authors:** Samuel Prince, Carl Munoz, Fannie Filion-Bienvenue, Pierre Rioux, Matt Sarrasin, B. Franz Lang

**Affiliations:** Département de Biochimie, Robert Cedergren Center for Bioinformatics and Genomics, Université de Montréal, Montréal, QC, Canada

**Keywords:** mitochondrial introns, group I, ERPIN, covariance models, infernal, RNA structure

## Abstract

Mitochondrial genomes—in particular those of fungi—often encode genes with a large number of Group I and Group II introns that are conserved at both the sequence and the RNA structure level. They provide a rich resource for the investigation of intron and gene structure, self- and protein-guided splicing mechanisms, and intron evolution. Yet, the degree of sequence conservation of introns is limited, and the primary sequence differs considerably among the distinct intron sub-groups. It makes intron identification, classification, structural modeling, and the inference of gene models a most challenging and error-prone task—frequently passed on to an “expert” for manual intervention. To reduce the need for manual curation of intron structures and mitochondrial gene models, computational methods using ERPIN sequence profiles were initially developed in 2007. Here we present a refinement of search models and alignments using the now abundant publicly available fungal mtDNA sequences. In addition, we have tested in how far members of the originally proposed sub-groups are clearly distinguished and validated by our computational approach. We confirm clearly distinct mitochondrial Group I sub-groups IA1, IA3, IB3, IC1, IC2, and ID. Yet, IB1, IB2, and IB4 ERPIN models are overlapping substantially in predictions, and are therefore combined and reported as IB. We have further explored the conversion of our ERPIN profiles into covariance models (CM). Current limitations and prospects of the CM approach will be discussed.

## Introduction

Sequencing of mitochondrial (mt) genomes (separately or as part of whole-genome projects) has become easy and affordable, but identifying and annotating genes in mt contigs often remains challenging. This is because mt genes, particularly in fungi, may contain a substantial number of (sometimes large) Group I and Group II introns, as well as difficult-to-recognize mini-exons that can be as small as three (Cummings et al., 1990b) or, at an extreme, a single nucleotide (Osigus et al., 2017).

In nuclear genome projects, the inference of gene models can leverage transcript alignments, in conjunction with alignments of conserved protein or structured RNA sequences from related species onto the genome sequence. However, mitochondrial transcript data are not only rarely available but also of limited help, as splicing of mt RNA precursors tends to be partial and is often difficult to interpret without expert manual intervention. Therefore, mitochondrial gene model inferences are usually only based on the set of conserved mitochondrial gene or derived protein sequences (Paquin et al., 1997; Gray et al., 1999; Lang et al., 1999; Burger et al., 2003; Lang, 2013). Evidently, this approach has serious drawbacks. When relying on publicly available sequences, these must be closely related to the genome to be annotated, and *a priori* be complete and accurate, otherwise implicit errors will occur *via* “community error propagation.” It is likewise possible to curate the gene annotations of neighboring species case by case, an approach that requires substantial input of a knowledgeable expert curator. Moreover, sequence matching of known coding or protein sequences (which is employed in both aforementioned approaches) can be fairly precise for delineation of larger exons, but can often fail for those smaller than ~30 nt, particularly when two or more small exons are “hiding” in long stretches of intron-rich sequence. It is here that high confidence and complete intron identification plays a crucial complementary role in revealing approximate locations of potential exons (i.e., in stretches of sequence between predicted introns). In addition, structural RNA inference of introns can provide clues on precise exon–intron boundaries flanked by conserved sequence features.

In the following we will first explain the distribution and general structural features of these introns, with emphasis on mt Group I and its sub-groups. Group II introns will not be further discussed as they are readily identified computationally, with a single search (Lang et al., 2007), based on two small adjacent helical regions (domains V plus VI). In stark contrast, Group I intron identification works very poorly with a general intron model and instead requires searching with models that represent the distinct sub-groups as well as structurally derived intron variants (Lang et al., 2007). We will then go on to explain two powerful search algorithms [ERPIN (Gautheret and Lambert, 2001) and Infernal (Nawrocki et al., 2009)] that are best suited for identifying these structured RNAs and their sub-groups, weighing advantages and potential drawbacks.

### Distribution and Structural Features of Group I and II Introns

Group I and II introns occur in a wide range of mitochondrial, chloroplast, eubacterial, bacteriophage, virus, and nuclear genomes [e.g., (Michel et al., 1982; Cech et al., 1983; Michel and Dujon, 1983; Shub et al., 1988; Ferat and Michel, 1993; Ohta et al., 1993; Turmel et al., 1993; Michel and Ferat, 1995; Qiu et al., 1998; Cho and Palmer, 1999). They are (or originated from) mobile elements that spread *via* intron-encoded proteins most notably “homing” endonucleases in Group I (Dujon, 1989; Henke et al., 1995; Belfort and Bonocora, 2014), and reverse transcriptases in Group II (Michel and Lang, 1985; Schäfer et al., 2003; Lambowitz and Zimmerly, 2004)]. In contrast to the eukaryotic spliceosomal introns in nuclear genes, Group I and II introns are characterized by elaborate, conserved (but unrelated) RNA structures that were first recognized in the early 1980s in fungal mtDNAs [e.g., (Dujon, 1980; Michel et al., 1982; Waring et al., 1982)]. Group I introns were shortly thereafter identified in ciliate nuclear rRNA genes and were demonstrated to self-splice *in vitro* without requiring protein factors (Kruger et al., 1982; Cech et al., 1983). This finding motivated a large number of research groups to investigate the “self-splicing” properties of Group I and II introns that were identified in their sequencing projects, to rather mixed results. Successful *in vitro* splicing in the absence of protein co-factors was reported for only a limited number of introns. More often, splicing depends on intron-encoded proteins [termed “maturases,” e.g., (Carignani et al., 1983)], or on proteins encoded in separate nuclear genes [e.g., (Kreike et al., 1987; Augustin et al., 1990; Bassi et al., 2002; Huang et al., 2005)]. In particular, mitochondrial introns turned out to be frequently unable to splice *in vitro* in the absence of protein co-factors [e.g., (Schäfer et al., 1991)], even under most un-physiological test conditions, like high salt, temperature, etc. (and, such negative results are evidently under-reported in the literature). Accordingly, the general notion that Group I and II introns are autocatalytic or self-splicing is quite misleading. Qualifying them as ribozymes, which in some instances undergo autocatalytic splicing *in vitro*, appears to be more in line with the published literature.

Mt. Group I introns were initially classified into Group IA, IB, IC, and ID [with an additional bacterial IE group added a few years later; for a review see (Hausner et al., 2014)], and further subdivided (e.g., IC1 and IC2). The initial mt group II intron subdivisions are Group IIA and IIB, later extended with the identification of a bacterial IIC (Zimmerly and Semper, 2015). Although reaching back as much as 32 years, this classification continues to be widely used and accepted. Group I classification is still based on the 87 available sequences at the time [see appendix in (Michel and Westhof, 1990)], collected from organelle and bacteriophage genomes, plus introns in ciliate nuclear rRNA genes. Notably, the vast majority of sequences came from fungal mt genomes, with more than one-third (38%) from a single species, *Podospora anserina* (Cummings et al., 1990a; Michel and Westhof, 1990). Evidently, this sampling is highly biased toward mt introns, and any of these groupings rely essentially on human expertise, rather than on computational methods. In the absence of a sufficient number of intron sequences per sub-group, which would have allowed a phylogeny- or sequence profile-based grouping, the initial ordering of group I introns into sub-groups gave most (but not all) weight to the P7 pairing, which is an essential part of the catalytic core of the ribozyme serving as a binding site for a guanosine cofactor (Michel et al., 1989). Other relatively well-conserved regions that were considered in addition are the P1 stem that defines the 5′ splice site and the P4-P5-P6 and P3-P7-P9 helices (Michel and Westhof, 1990). Note that substantial variation of the P7 motif was accepted within given sub-groups as long as overall structural or sequence relatedness was recognized, which speaks against the popular characterization of intron groups *via* P7 sequence motifs (“logos”). In fact, logos emphasize the most predominant primary sequence, therefore lack detail on sequence and structural variation (i.e., the characteristic helix-bulge-helix of P7) that is essential for ribozyme catalysis.

### Computational Methods for Intron Identification

Basic similarity search algorithms, as implemented in BLAST and FASTA are woefully inadequate in identifying introns for two reasons. The first being relatively high levels of sequence variation in introns, which can degrade the quality of high-scoring sequence pairs, and thus lead to imprecise and fragmented hits. The second reason is that similarity comparisons are blind to secondary structure, which limits their capacity to bridge distant conserved motifs. Instead, probabilistic approaches using sequence profiles (based on structured alignments of multiple sequences, including information of secondary structure pairings) are required to spot the regions of similarity that are small and spread out over intron sequences that can reach up to 7 kb [e.g., (Liu et al., 2020)] and beyond. Currently available and popular search algorithms are ERPIN (Gautheret and Lambert, 2001) and Infernal (Nawrocki et al., 2009). ERPIN is based on column-wise computation of probabilities at the nucleotide and structure level, focusing on the detection of distinct conserved sequence motifs and helices in given structured sequence alignments (to be supplied by the user). In contrast, Infernal leverages the HMM approach, computing emission (at a given column) and transition probabilities (from one column to the next), but applies covariance modeling (CM) as a second layer search mechanism to initial HMM hits. The CM architecture is a stochastic context-free grammar (SCFG) profile which, in the same spirit of HMMs, consists of states (with emission and transition probabilities) associated with the single nucleotides and pairs that make up the RNA structure. CMs are therefore expected to be more sensitive than ERPIN, and because of the underlying HMM approach that in contrast to ERPIN allows for insertions and deletions that are not identified as such in the search model, useful in improving structured sequence alignments.

In 2006, 16 years after the initial Group I intron classification by Michel and Westhof, the increased number of available organelle genomes (then the most substantial and diverse source of intron sequences) allowed the development of intron search models for automatic identification and classification of virtually all known organelle group I and II introns with high confidence (Lang et al., 2007). The underlying algorithm for this approach has been **ERPIN** (Gautheret and Lambert, 2001). Yet even in 2006, the low number of sequences in some intron sub-groups had limited automated approaches with ERPIN because a computationally objective confirmation of intron group consistency was out of reach. As a consequence, structured sequence alignments may have in rare instances included a sequence from an unrelated sub-group, potentially leading to intron predictions in both the target and contaminant sub-groups. Notwithstanding, the use of these ERPIN search models has been reasonably precise and complete (Lang et al., 2007), which was a requirement for developing our MFannot mitogenome annotator.^1^

Since 2006, no systematic update of our intron sequence alignments has been conducted to verify the ERPIN approach and the findings. At the algorithmic level however, the development of **covariance models** (**CM**; Nawrocki et al., 2009) have become an attractive alternative to ERPIN, due to a recent substantial performance increase (Nawrocki et al., 2009; Nawrocki and Eddy, 2013), resulting in search times comparable to, if not better than, ERPIN. In fact, CM analysis has enabled detection of the widespread presence of group IA3 and IB4 introns in Archaea (Nawrocki et al., 2018). The CM approach has not yet been compared against ERPIN, or more broadly verified for both its sensitivity and its precision in sub-group classification. Incidentally, a recent study had leveraged CMs uniquely in the context of mitochondrial group ID introns, limited in scope to both the core motifs as well as to the relatively narrow lineage of pezizomycete fungi (Cinget and Bélanger, 2020). Furthermore, the aligned ID intron sequences were taken from the now defunct GISSD intron database (Zhou et al., 2008), which implies the quality of the underlying data must be taken for granted. Thus, the specificity and sensitivity of the resulting CM to the ID group remain to be clarified.

### Challenges in Assembling a Consistent Set of Group I Intron Predictors

The currently available approaches for modeling RNA sequences with 2D structure layered on, ERPIN and Infernal/CM, have both specific advantages and drawbacks. The strength of ERPIN is in examining clearly defined structural or sequence motifs, by providing the user with the option of identifying distinct motifs and searching them in any given combination and order. The ERPIN search strategy can be optimized to be both sensitive and rapid in execution (despite lack of parallelization), by searching highly conserved motifs at an initial level (preferentially single-stranded region that are much more rapidly identified than helical interactions), and followed by inclusion of other peripheral motifs. It is important to realize that this motif-driven approach allows for modeling of pseudoknots, which in Group I introns include the universal P7 structural motif—a crucial element of the ribozyme catalytic domain (Michel and Westhof, 1990). ERPIN requires that conserved motifs, such as P7, be supplied together with a structural multiple sequence alignment, which can often be a challenging task. Another clear drawback of ERPIN vs. Infernal/CM is its unforgiving rigidity in defining a search model with distinct sequence or secondary structure motifs. For instance, nucleotide deletions in helical regions of search models are not allowed, contrary to the CM approach, which also accepts and then properly aligns nucleotide insertions and deletions (indels) of the resulting hits. In addition, partial hits will not be reported by ERPIN, which is an issue with derived intron structures that carry shortened or completely lacking motifs. Finally, too much sequence variation of a target ncRNA may result in ERPIN models that produce few or even no results. A solution proposed by the authors is a subdivision of sequences and respective ERPIN models, a “divide and conquer” strategy that we already successfully employed with our initial set of ERPIN intron predictors (Lang et al., 2007).

**Infernal** (cmsearch) on the other hand does report partial hits, and has substantially better sensitivity in sequence motif identification, as it uses an **HMM-SCFG approach** of assigning emission and transition probabilities (rather than the column-wise probabilities of ERPIN). This may be relevant as the current implementation of CMs proscribe strict processing from 5′ to 3′ of the given model, thus treats pseudoknots only at the primary sequence level. Yet, as long as the pseudoknot motif has significant nucleotide sequence conservation (which is not necessarily the case for Group I introns), the increased sensitivity of the HMM approach may (or not) compensate for the lack of pseudoknot helix modeling. Additionally, and in contrast to EPRIN, cmsearch excels at finding matches with CMs containing only few aligned sequences. The execution times of cmsearch may be faster than equivalent ERPIN searches, depending largely on given search models and the available CPU, as cmsearch natively supports parallelized computations. Taken together, CM alignments are attractive for the expert development of alignments because of its flexibility in finding matches and because of the formatting of results as a structured alignment. To be clear however, this functionality does not liberate the user from providing an initial multiple sequence alignment together with a 2D structure line. For this, a pre-alignment at the sequence level with one of the many multiple sequence alignment tools [e.g., Muscle (Edgar, 2004)], followed by prediction of secondary structure pairings [e.g., RNAalifold (Bernhart et al., 2008) or R-scape (Rivas et al., 2020)] will provide a structured alignment that still needs to be refined by an expert.

### Short Term and Long-Term Objectives

The unprecedented number of mt genomes that have been added more recently to the GenBank repository has progressed from a severe lack of sequence data to “land of milk and honey” with regard to intron analysis. In this paper, we will focus on the 662 fungal mitochondrial (mt) DNAs, identified in various sections of GenBank by November 2021 (see below), because of their most elevated intron density [e.g., 81 in *Endoconidiophora resinifera*, (Zubaer et al., 2018)], broadly covering all but the more recently identified nuclear and bacteria-specific sub-groups. Our objective for intron model building is automated alignment of well-conserved and universally present motifs in currently defined intron sub-groups, starting as a test case with mt Group I introns [i.e., as originally defined in the seminal Michel and Westhof publication (Michel and Westhof, 1990)]. The resulting structural models will be tested for overlapping predictions, either for dismissal of traditional sub-groups or the inference of additional ones. The questions that we will address in the context of intron identification and classification are as follows:

- is the currently accepted intron sub-grouping for IA, IB, IC, and ID valid and consistent from an evolutionary/computationally point of view;
- does automated, probabilistic intron classification with ERPIN identify known fungal mt introns within the given sub-groups and without ambiguity.

We will conclude with a brief preview on covariance searches with Infernal/CM, to test whether CMs are as performant in intron identification, and as suitable for intron sub-grouping as ERPIN.

## Materials and Methods

### Building of ERPIN Intron Models

As a starting point, aligned intron sequences for each sub-group that are listed in the Michel and Westhof publication (Michel and Westhof, 1990) were shortened to the virtually ubiquitous helical regions P2,4,6,7,8 and adjacent conserved single-stranded sequences, and a respective 2D structure line in ERPIN format was added to the sequence alignments. The resulting models were searched against the set of 662 fungal mtDNAs using the ERPIN (version 5.5.4) wrapper script RNAweasel that allows to eliminate identical as well as closely related sequences in the aligned training set. The aligned matches were added to the current ERPIN model to create an extended ERPIN model, visually inspected for potential misalignments, manually corrected or adapted (i.e., by extending the size of variable insert regions that separate the conserved sequence motifs), or otherwise discarded if inconsistent with the overall alignment. After repeating this process several times, the consistency of the resulting model was tested by searching against the Michel collection of sequences, expecting to match only members within the same intron group, or at most identifying conflicting matches with low scores. The same type of test was conducted with sequences from the GISSD database, and finally, the results of all our searches with different ERPIN models were checked for conflicting matches.

### Identification of Conflicting Matches With Different ERPIN Models

Conflicting matches were identified with a Python script that analyses the coordinates of hits across multiple RNAweasel log files, to flag intron intervals that are predicted by more than one ERPIN model. First, conflicting hits (i.e., sharing at least 1 nt between their corresponding genomic intervals) were assigned to a group. For each group, the proportion of shared conserved nucleotides (in capital letters in the log file) between the hits was computed to aid in the separation of the hits into the two categories “conflicting prediction” or “overlapping introns.” The final parsing of the result was done manually; hits that shared the same (or almost the same) start and end position were labeled “conflicting” (>95% identical conserved positions found by both models) while the rest of the hits were identified as “overlapping introns.” For the IB sub-group analysis, the parsing was done automatically without distinction between “conflicting” and “overlapping introns.”

### Development of ERPIN Search Strategies

Finding an optimal search strategy for every given ERPRIN model is essential for execution speed, sensitivity of searches as well as appropriate cutoff values. According to previous experience search strategies with three (rarely four) search levels are most effective (using-add statements as described in the ERPIN manual). The initial search level will pinpoint potentially intron-containing genomic regions, with subsequent search levels selecting those that meet the full set of constraints (for more details on the principals of element regrouping and order of search levels, see the main text). Initially, Skylign (Wheeler et al., 2014) is used to generate a logo of a multiple sequence alignment (MSA). Skylign converts the MSA to a Hidden Markov Model (HMM) in order to estimate position-specific (including gaps) probability distributions, or logo stack heights. The letter proportions per stack (or, position) are computed from the respective estimated nucleotide probabilities. Once the logos are obtained, an in-house script computes the average stack height across each distinct motif (e.g., segment of single-strand, or segment half of a paired sequence). Motifs with higher average probabilities are identified and labeled according to position on the secondary structure line. Motifs with lower average probabilities are subsequently defined. Finally, the script combines the motif definitions, along with cutoff scores derived from the (ERPIN) tstat summaries of the respective motifs, to automatically create optimized parameters for an intron model search.

### Conversion of ERPIN Model Alignments Into CM-Compatible Stockholm Format

ERPIN models have a custom-encoding of the structure line information (based on consecutive numbering of elements; if a number occurs only once, it is a single-stranded region; if the number occurs twice, it defines a helical interaction). In order to permit the use of the ERPIN model information for building and calibration of respective CMs, the ERPIN format needs to be converted into Stockholm format, which uses bracket expressions for identification of helices, but requires additional encoding with pairs of upper/lower case characters (known as WUSS shorthand) to identify the pseudoknot that is present in most ERPIN intron models. Structure lines containing a pseudoknot, and modified by converting the helical components to WUSS shorthand (AA..aa), will be recognized by Infernal tools and interpreted only at the nucleotide conservation level.

## Results and Discussion

### Collecting and Formatting All Publicly Available Fungal mtDNAs—A Non-trivial Task

The success of our project to update Group I and II intron search models critically relies on the availability of a taxonomically broad and complete collection of fungal mtDNA sequences. The National Center for Biotechnology Information (NCBI) sequence databases have continued to grow exponentially, including the Organelle Genome Database (OGD) that housed as many as 12,582 mitochondrial genome assemblies as of October 19th, 2021. While the OGD appears to be the NCBI’s front-end to searching mitochondrial genome records, assemblies can also be found in the NCBI Genome Reports (GR) section and in the continually updated RefSeq release of mitochondrial records. Searches against the Nucleotide (NT) database reveal again additional mitochondrial assemblies of varying quality and completeness. There are several issues with the vast databases of NCBI that hamstring studies aimed broadly at fungi. The first issue is that the fungal lineage represents only a minority of records across all databases, as shown in Supplementary Table S1. The number of fungal records typically make up less than 5% of the total, with the exception of the NCBI Genome Reports, after taking into account redundant accessions. The second issue is related to inconsistencies in accessions listed across the above-mentioned databases. As shown in Supplementary Figure S1, accessions listed under the GR appear to be completely contained within the OGD, but there is a handful of records uniquely found in RefSeq, and more than 1700 unique to NT, which can introduce a large sample size bias. The last issue is related to gaps in the GenBank records themselves. Full taxonomic, annotation, and sequence information is typically bundled in GenBank records, but some records, mostly from the OGD and GR, are missing the underlying genomic sequence. Furthermore, almost all of the incomplete records are the same in both the OGD and the GR, whereas a minority are incomplete in the curated RefSeq collection (Supplementary Figure S2). Ironically, the largest database of GenBank records, NT, has the least number of incomplete records.

In order to assemble a taxonomically broad collection of fungal mt genomes, we extracted records from the OGD^2^ and GR (see Footnote 2) front-ends and then combined into a preliminary list. The NT collection (in compressed fasta format) was then downloaded^3^ and filtered for keywords related to partial or complete mitochondrial genome assemblies. The resulting accessions are parsed and added to the OGD and GR collection. A python script, leveraging the BioPython modules (Cock et al., 2009), was written to parse GenBank files and output a clean fasta nucleotide sequence record for each mitochondrial contig (i.e., disregarding information on gene, exon, and intron positions), with a header formatted to include a short unique ID (derived from genus, species, and accession) in the first field, followed by full genus and species name, then full taxonomic information, and capped with the original accession. Such a format is more conducive to careful selection of species diversity, and visual inspection of phylogenetic trees given how current phylogeny tools function.

### Automation of ERPIN Search Strategies, and Phylogenetic Filtering of Structured Alignments

Since searches involving multiple motifs may quickly become demanding if not unfeasible in terms of CPU time and memory usage, ERPIN provides the option of a **multi-leveled search strategy** (Gautheret and Lambert, 2001; Lambert et al., 2004). It allows grouping of (sequence or structural) motifs that are searched in iterative steps. The initial search level will pinpoint potentially intron-containing genomic regions, with subsequent search levels selecting those that meet the full set of constraints. In other words, the execution speed depends essentially on the choice of the first-level motifs—preferentially well-conserved single-stranded regions that are most rapidly identified. Yet, finding the best search strategy requires a rather lengthy trial and error optimization by the model developer—motivating the development of a more objective computational procedure (Auto-strategy; in-house script available on request). It takes an ERPIN model (i.e., a set of aligned sequences with secondary structure predictions) and a collection of targeted genome sequences as an input and constructs a search strategy with corresponding cutoff values for the given ERPIN model and sequence collection. More specifically, the algorithm (for details see Methods) allows the computation of a three- or four-level search following several principles: (1) selection of closely spaced and strongly conserved sequence motifs for level one, to enable speedy initial searches; (2) at all levels, combination of several conserved motifs, sufficient to avoid false positives; and (3) a final addition of the remaining elements. Auto-strategy often results in more effective and specific searches compared to manual strategies and may serve as a quickly computed starting point for further optimization by the model developer. The tests carried out during the development of Auto-strategy were performed on sub-groups A1, A3, B, C1, C2, and D of mitochondrial group I introns. The number of hits obtained by the automatic strategies was frequently higher compared to the manual ones. Yet, subsequent manual finetuning of the Auto-strategy often led to further improvements.

### A Computational Approach to Validating Group I Intron Subdivisions

**Figure 1:**
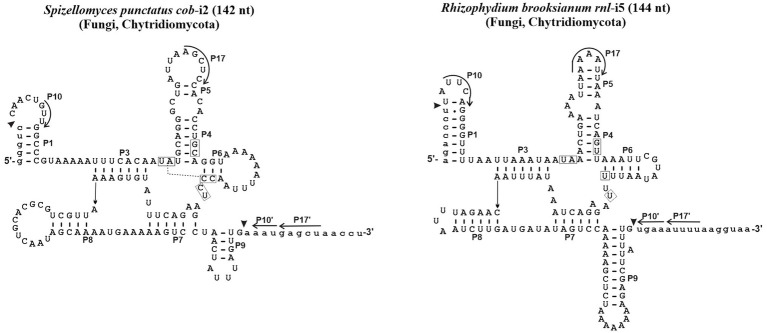
Examples of derived Group I intron structures. With only 144 and 142 nt, the *Spizellomyces punctatus cob* intron 2 and *Rhizophydium brooksianum rnl* intron 5 do contain the universal intron core structure, but lack overall sequence conservation, are rich in A + U, and occasionally lack peripheral domains and typical interactions. Like other unusual mt introns in these two fast-evolving species, introns do not splice *in vitro* and only a small fraction (e.g., 3 out of 15 in *S. punctatus*) is identified with the ERPIN models IA1, IA3, IC1, IC2, IB, IB3, and ID as described below.

Since 1990, no computational study has been conducted to verify the validity of the initial assignment of Group I introns into sub-groups (Michel and Westhof, 1990). Indeed, the set of ERPIN models that we built in 2007 essentially relied on the validity of these groupings (Lang et al., 2007), although omitting a distinction between sub-groups IB1, IB2, IB3, and IB4 due to substantial overlaps in predictions. In addition, we then created a “Group I, derived” sub-group for those introns that were not identified with the regular ERPIN models. In other words, derived introns do not well fit with typical consensus sequences and occasionally lack the peripheral structural domains and interactions. Two examples are presented in Figure 1. ERPIN models that identify the derived introns contain only subsets of the Group I core elements.

Whereas this computational intron group identification procedure has been unmatched and widely used for mt gene model inference (see Footnote 1), it is not without potential pitfalls. Its ERPIN models could not be rigorously tested for confident resolution of conflicting hits due to a lack of intron sequences at the time. Thus, using the best E-value as an *ad hoc* criterion may occasionally lead to misidentification when model predictions overlap. Secondly, contamination of ERPIN models with sequences between closely related groups (e.g., IC1 and IC2) was difficult to identify and avoid, given the small number of available sequences. Thirdly, a large fraction of group I introns contains long intron ORF insertions of more than 1,000 nt, whereas others are short and compact. The underlying intron alignments of the ERPIN models are therefore long, yet have to predict a fraction of relatively small introns. This introduces the opportunity for matches across exon boundaries. For instance, a single hit might start at the 5′ portion of a small intron and incorrectly match the 3′ of a separate, downstream intron which would effectively “bury” the bridging exon. The proportion of such potential misidentifications is estimated to be low (a few percent), but has not been rigorously quantified. It also remains to be seen in the context of proposed nested introns, or “twintrons” (Hafez and Hausner, 2015; Mukhopadhyay and Hausner, 2021), that may be more frequent than currently assumed. In fact, conflicting ERPIN predictions may be a way of pinpointing candidate twintrons.

### An Updated Set of Group I Intron ERPIN Models

**Figure 2:**
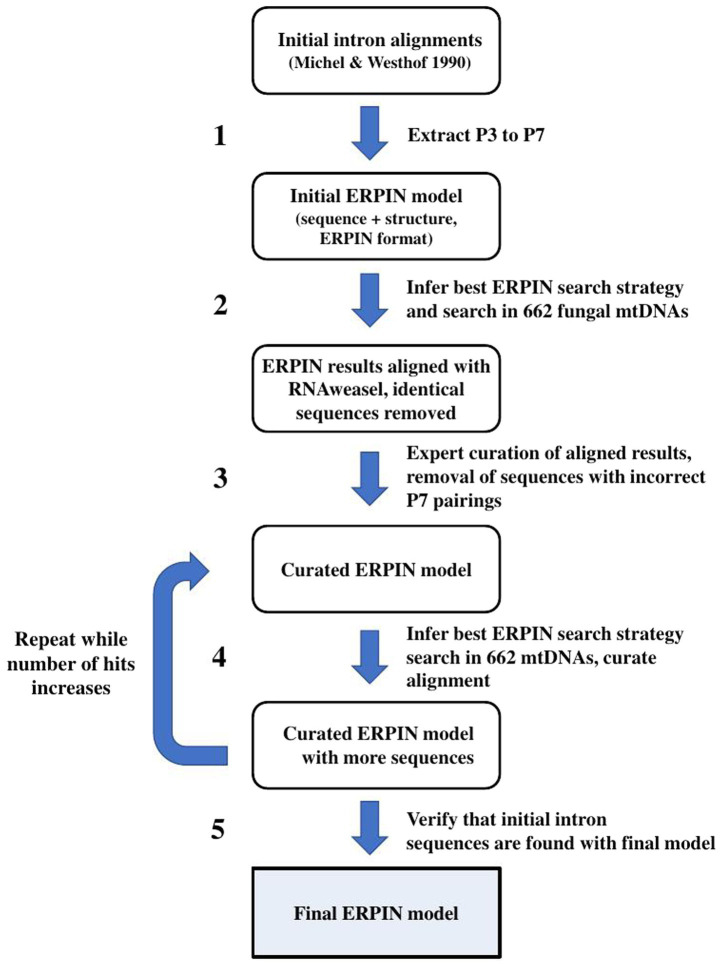
Iterative ERPIN model construction. The procedure was applied to all separate intron sub-groups.

**Figure 3:**
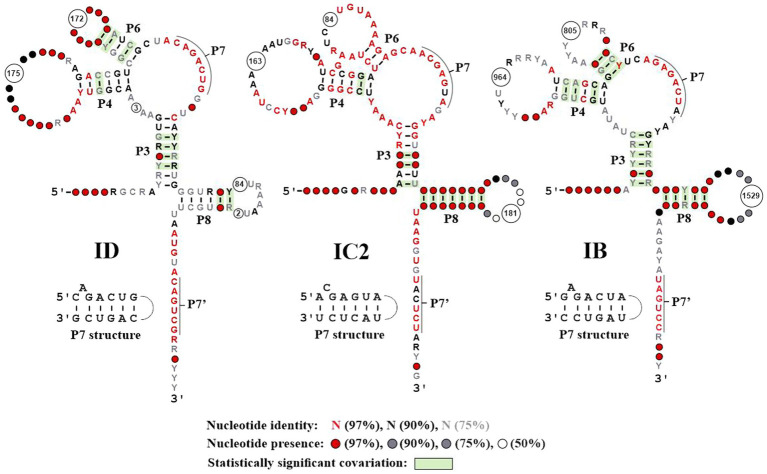
Conserved features of ERPIN models. The three images correspond to the secondary structure drawings produced by R-scape (Rivas et al., 2020), when providing structured alignments of ERPIN models ID, IC2, and IB (the most productive in terms of numbers of hits) as input. The graphs are colored according to R-scape’s conventions. As R-scape removes columns with less than 50% nucleotide presence from the graph, the number of removed columns are identified in the consensus structure as a circle with the number of removed columns inside. The P7 pseudoknot is annotated in the graph as two single-stranded regions, marked by a black line. The corresponding helical pairing is presented in a separate drawing. The number and structure of conserved elements in the remainder of ERPIN models IA1, IA3, IB3, and IC2 is essentially the same as depicted in this figure (not shown).

To address the above questions, we built a new set of ERPIN models (Figure 2) starting from the 87 sequences used in the originally defined Group I intron subdivision [named “Michel collection” in the following; (Michel and Westhof, 1990)]. After building structural alignments (ERPIN models) comprising P3 throughout P7 pairings (Figure 3), including conserved flanking sequences (if present), the set of 662 fungal mtDNA sequences (see above) was searched. Best hits were selected and added to the initial model alignment to improve model sensitivity (for details see Methods). The total numbers of predicted introns are listed in Table 1, together with conflicting predictions. In all instances, there was a large E-value difference between conflicting predictions, which allowed unambiguous intron group assignment.

**Table 1:** Number of intron predictions for distinct Group I sub-groups (in 662 mtDNAs).

	IA1	IA3	IB	IB3	IC1	IC2	ID
Total	960	78	3,582	202	337	948	1,105
E-values (e-)	(9–35)	(16–42)	(4–39)	(14–44)	(22–94)	(27–95)	(12–44)
Conflicting (true)	3(IB)	-	88(IC1)	-	2(IC2)	-	-

**Figure 4:**
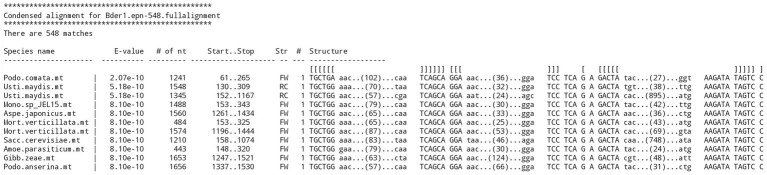
RNAweasel output of ERPIN search results. Example of a formatted output from a “derived ERPIN model,” demonstrating sorting of results by E-value, marking of conserved motifs in a structure definition line, marking of conserved, searched sequence blocks in upper case, and indication of the length of variable regions.

Note that the process of building increasingly broader and detailed structural alignments depended on a program named RNAweasel,^4^ which extends the functionality of ERPIN. RNAweasel aligns the resulting matches against the search model in the format of a new ERPIN model that can be directly used for subsequent searches or merged with previous alignments. In addition, it has functionalities, such as ordering of hits by E-value, removal of sequences from the alignment that are either identical, or closely related, using a phylogenetic distance measure. Finally, the RNAweasel output helps with the evaluation in providing a structured view of the search results (for an example, see Figure 4).

An inspection of the updated ERPIN models shows few conflicting predictions (i.e., covering the same genome loci with nearly identical coordinates), without any conflict observed for Group IA3, IC2, and ID. A small number of overlaps were observed between models IC1 and IC2 (0.6% of IC1 hits are IC2 introns), IA1 and IB (0.3% of IA1 hits are IB introns), and IB and IC1 (2.5% of IB hits are IC1 introns). Yet, in every case, a large difference in E-values allows a clear identification of the best-fitting intron sub-group. A special case of conflicting predictions is “**overlapping introns**” where two introns are identified in the same region but have different predicted upstream and/or downstream splicing sites (reported in Table 2). Again, the number of conflicts is small, occurring in only 0.7% of all total predictions. 97% of these cases are between the IB or IB3 models and other sub-groups (Table 2). An inspection reveals that in the majority of instances, the shorter of the conflicting alternatives is the one that is consistent with MFannot gene models.^5^ In any case, we suggest that ERPIN intron predictions or MFannot gene models should be inspected by an expert before use in publications.

**Table 2:** Conflicting partial intron predictions.

	IA1	IA3	IB	IB3	IC1	IC2	ID
IA1	0						
IA3	1	0					
IB	28	14	4				
IB3	0	19	6	0			
IC1	1	0	14	0	0		
IC2	0	0	13	0	0	0	
ID	1	0	2	0	0	0	0

In the following sections, we will describe and discuss Group I models in the order ID, then IA1, IA3, IC1, and IC2, and finally IB with its four sub-groups. Starting out with the ID models will establish a basis for an unambiguous discussion on variations in the P7 motifs conservation, without ambiguity, as the result of ID searches do not conflict with any other Group I search, except for three instances of partial intron predictions (Table 2).

### Structural Conservation of Group ID Introns

**Figure 5:**
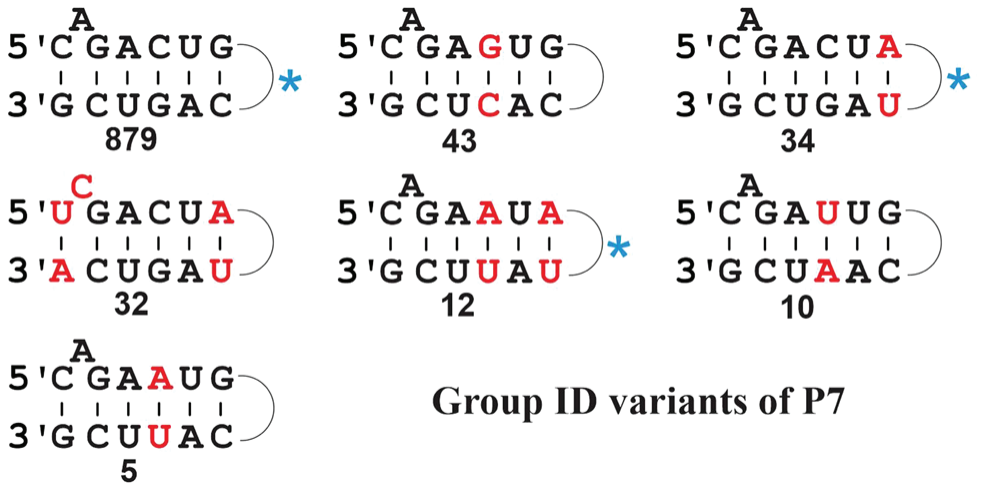
Frequency of P7 structure variants in fungal mt Group ID introns. The numbers are compiled from the search result with the updated ID ERPIN model (see main text), identifying a total of 1,107 hits (span of E-values from 7.17e-44 to 4.94e-11); none of the respective intron sequences was identified with other group-specific searches, and all are therefore considered *bona fide* ID introns. Variant motifs with at least five identical P7 matches (numbers indicated below the structures) are listed, and nucleotides that differ from the most prevalent motif are marked in red. The most prevalent P7 motif (879 hits) is 5’-C_A_GACUG…CAGUCG-3′. The blue asterisk beside motifs indicates listing of the same motif in the Michel and Westhof compilation (Michel and Westhof, 1990).

**Figure 6:**
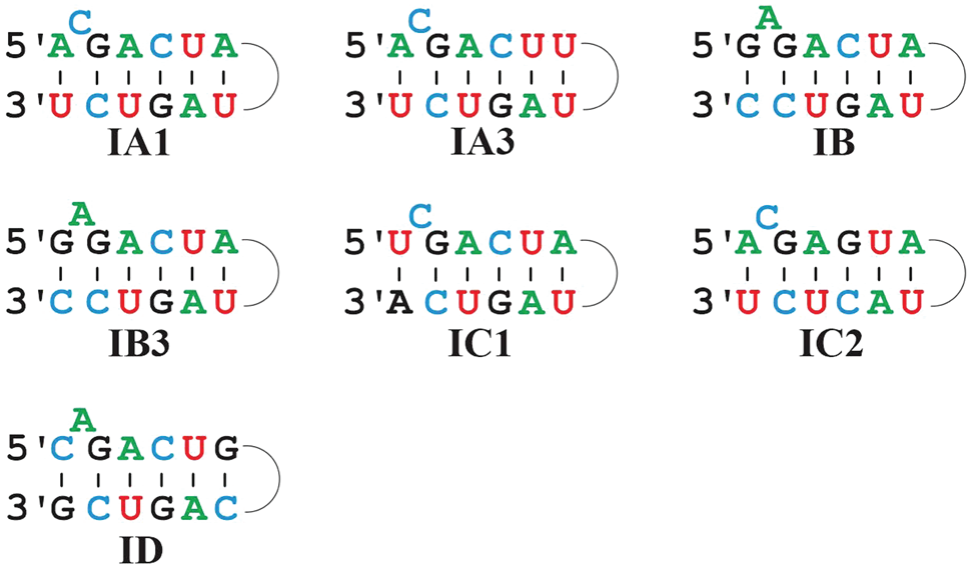
Comparison of predominant P7 motifs in mt Group I intron sub-groups. Most frequent P7 motifs in mt Group I intron sub-groups. Note that IB and IB3 have the same P7 motif, but that they are clearly distinct otherwise and identified with separate ERPIN models. IA3 introns have a frequent alternative motif A_C_GACUG … UAGUC_U (i.e., with a single nucleotide difference to the one depicted above).

Group ID comprises a sizeable collection of mt introns (1,105, when searching against our set of 622 mtDNAs) with a predominant P7 structure 5-C_A_GACUG --- CAGUCG-3′ (Figure 5, upper left; for a comparison of P7 motifs among various groups, see Figure 5). Yet, searches also identify sequences with various different P7 motifs (Figure 5) that come with E-values well within the range of canonical ID introns. Among these variants, 32 have a most unusual P7 motif containing a bulged C residue (Figure 5; middle, left), which happens to be the predominant P7 structure of mt IC1 introns (Michel and Westhof, 1990). These 32 variant ID introns are not found with the mt IC1 ERPIN model, suggesting that the P7 structure alone is a potentially misleading feature when used for intron group identification. To exclude the possibility that our ID ERPIN model may contain sequences from other sub-groups, thus leading to potential misidentification, only those sequences with the predominant P7 structure were used to build a new ERPIN model. However, when searched against our mtDNA collection results were essentially the same, finding all variant P7 sequences listed in Figure 5, and within the range of E-values as introns with the canonical P7 structure (Figure 6). A phylogenetic analysis with all ID intron sequences is overall not well resolved (not shown), but regroups some of the ID intron members with variant P7 structures, suggesting an evolutionary process that transitions from one to another evolutionary stable P7 conformation.

Note that the total number of introns listed by P7 variant in Figure 5 does not add up to a total of 1,105 hits. The missing 92 sequence have either variant P7 motifs not listed in the figure, or after manual inspection, the predicted sequences fail to form a proper P7 pairing. This is due to a rare ERPIN misidentification where the absence of a well-fitting match reports a spurious alternative. Despite issues with the prediction of a P7 motif in these few instances, these hits suggest the presence of true introns and are thus of some value for the inference of gene models. There are several potential reasons for this type of error, nonetheless. For instance, sequence or genome assembly error may be responsible, or presence of intron structures that exceed the length of the respective ERPIN models, or genetic rearrangements due to intron mobility that may introduce sequence duplication and recombination with other intron sub-groups. At about 0.7% of the total count, this constitutes a tolerable degree of uncertainty. Yet, it reminds us that intron identification is but another tool to provide evidence for the detection and resolution of inconsistencies, as part of a more complete gene modeling procedure.

### Distribution of Group IA1, IA3, IC1, and IC2 Introns

In contrast to sub-group IA1, IC1, and IC2 introns that are frequently identified in mtDNAs (Table 1), sub-group IA3 introns are remarkably rare. In fact, the Michel collection only contains four plastid IA3 introns and no mt representative. It was therefore surprising to find as many as 78 strong mt hits (in the E-value range between 4.71e-16 and 1.81e-42) distributed across Fungi, but with a strong preference for basidiomycetes. Notably, 18 of the 78 sequences do not have the predominant bulged C in the P7 motif but instead an A, and these sequences closely regroup in the less specific e-16 range of E-values. When separating sequences in the IA3 ERPIN model by these two P7 motifs to form two distinct sub-models, these identify different but somewhat overlapping sets of introns. If this result may be taken to suggest a separate, new (IA4?) sub-group remains to be clarified, once more mtDNAs become available.

Despite some structural similarities, sub-group IC1 and IC2 ERPIN models are clearly distinct in terms of mt intron identification, with only marginal conflicts. The few conflicting predictions are separated by substantial E-value differences, thus allow for unambiguous sub-group assignment. In this context, it is interesting to test entries in the GISSD database, which lists a total of 1789 sequences, with close to half (837; most in nuclear eukaryotic rDNAs) labeled as IC1. Curiously, Group IC1 entries in the database do not list any of the mt IC1 introns in the Michel collection that served as our starting point for developing an mt IC1 ERPIN model. When searching the Michel collection with the IC1 model it identifies all (and only) mt IC1 entries. It was therefore interesting to test if the mt ERPIN model would also identify introns in nuclear rDNAs (at such a large evolutionary distance), and that despite a strong difference in nucleotide bias (mt sequences most A + T rich, vs. nuclear sequences G + C rich). The results show clear identification of 479 out of the 837 listed IC1 introns, with E-values ranging from 5.40e-25 to 6.53e-49, and without modification of the ERPIN search parameters. The identification of the remainder of nuclear IC1 introns was possible only after transitioning to a nuclear-sequence specific ERPIN model, however. Evidently, these two lineages of introns (mt vs. nuclear) have undergone a separate evolutionary path, under different genetic constraints.

### Confirmation of Group IB3, yet No Computationally Distinct IB1, IB2, and IB4 Sub-Groups

**Figure 7:**
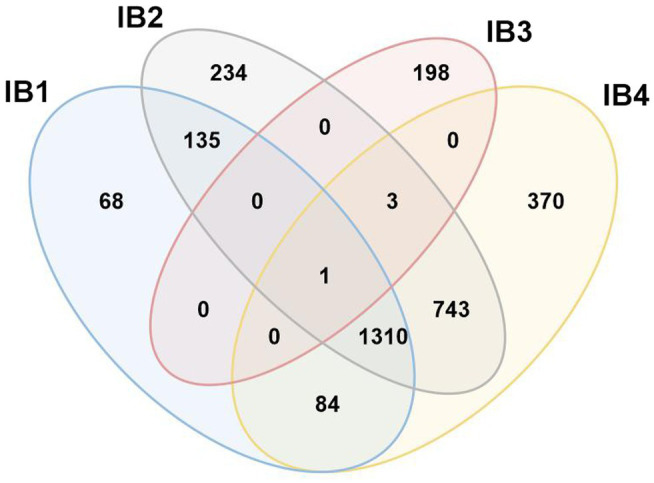
Venn diagram demonstrating conflicting hits among IB sub-groups. Search results with ERPIN models specific for the four Group IB sub-groups [as proposed in (Michel and Westhof, 1990)] were analyzed for conflicting hits, with respective numbers displayed in a Venn diagram. With the notable exception of IB3 which has only 4 conflicts out of a total of 202 intron sequences, IB1,2 and 4 overlap substantially in identification, without a possibility to distinguish by E-value. We therefore combine IB1,2 and 4 into a formal group IB that will co-exist with a separate IB3.

In an attempt to follow up on the proposal of four separate IB sub-groups, ERPIN models were developed according to our protocol and tested for potentially conflicting predictions. Whereas IB3 turns out to be a small, clearly separate group, the other three sub-groups overlap substantially in predictions (Figure 7), without a possibility for separation based on E-values. The IB1, IB2, and IB4 models properly identified representatives in the Michel collection with high scores, confirming the validity of the model building procedure. Yet, searches against the GISSD intron collection resulted in matches with barely recognizable distinction between the sub-groups. We conclude that IB1, IB2, and IB4 are too closely related for establishing distinct sub-groups, and have therefore joined them into a IB super-group as reported in Tables and Figures above.

### Cm Analyses Based on ERPIN Structural Alignments

A final point of interest is the conversion of ERPIN models into covariance models, for comparing the performance and precision of the two conceptually very different approaches. From a technical point of view, this conversion is easy as the alignment in ERPIN models is in fasta format, which is readily reformatted into Stockholm format (.sto) required for cmbuild [infernal; (Nawrocki et al., 2009)]. Because the ERPIN intron models contain pseudoknots that are encoded by a numbering scheme, the respective structure line for the sto format was translated in WUSS format. The current Infernal version does not use pseudoknot information and treats pseudoknot pairings as conserved at the nucleotide level only. For testing purposes, we chose two ERPIN models, ID and IA3 that differ substantially in length and in the degree of relatedness among the aligned sequences. The ID alignment is relatively short (560 nt positions), compact, and moderately conserved, whereas IA3 is long (3,042 nt), with very large insertions (up to 2011 nt) and highly conserved. The outcome is disappointing in the sense that both CMs have a smaller number of complete matches (776 for ID; 45 for IA3) compared to ERPIN model searches. The total numbers of matches (better than 1.0e-2) with CMs is substantially higher than the total number of respective ERPIN matches, indicating a better potential to identify more introns, although missing the ability to properly align the complete CM to the genome sequences. To our interpretation, the CM approach would profit from reorganizing the search algorithm, from a strictly HMM-like scanning from 5′ to 3′ toward a modular motif-driven approach used by ERPIN—which may at the same time resolve the issue of introducing pseudoknot information in CM searches.

## Concluding Remarks

Here we present an update of our previous work (Lang et al., 2007), and a more detailed description on the identification of mt Group introns, in light of new publicly data available, using ERPIN models. The update makes progress on some remaining issues from the previous work, extends the accuracy of the models, and sheds light on Group IB introns. Enhanced model sensitivity and specificity was achieved through two means. First, multiple sequence alignments of intron sequences were significantly extended by virtue of newly available data. Second, we developed a more systematic approach to curation of alignments, to exclude sequences that do not belong with a given sub-group (increased risk of incorrect identification). While an overall increase in model sensitivity was achieved, sub-groups IB1, IB2, and IB4 were found to be too closely related, which hampered model specificity, suggesting that IB1, IB2, and IB4 may be dismissed as sub-groups. On the other hand, Group IB3 introns were found to be sufficiently distinct to build a highly sensitive and specific ERPIN model. The current intron predictors are expected to improve the gene modeling of the MFannot tool as well as provide more precise structural intron information.

A remaining gray zone of Group I intron identification pertains to those that appear less well-structured, or “derived.” Our attempts to establish clearly distinct ERPIN models that would include those derived introns have been so far without success. Likewise, our attempt to transition from ERPIN to expectedly more sensitive CM searches has come with mixed success. It has provided more hits than ERPIN, however at the cost of a reduced number of full intron hits and a large portion of partial hits with borderline scores. To our assessment, a modification of the CM approach that allows modular search of conserved motifs or regions might be a potential solution, which would at the same time allow for the use of pseudoknot information.

Evidently, a continued search for additional distinct ERPIN sub-groups would be in order, but its algorithm is of little help with developing new structured alignments as required. For this, a more modular Infernal version in combination with primary sequence alignment [e.g., Muscle (Edgar, 2004) in combination with HMM searches (Eddy, 2011)] with secondary structure modeling [e.g., RNAalifold (Bernhart et al., 2008) or R-scape (Rivas et al., 2017)] would be preferable.

## Data Availability Statement

The original contributions presented in the study are included in the article/Supplementary Material, further inquiries can be directed to the corresponding author.

## Author Contributions

SP investigated conflicting intron predictions. CM was in charge of covariance analyses and the design of R-scape graphics. FF-B was involved in developing ERPIN search strategies. PR designed and coded RNAweasel. MS provided informatics and coding support. BL established optimized ERPIN models. All co-authors participated in discussing and writing the manuscript. All authors contributed to the article and approved the submitted version.

## Funding

The authors acknowledge generous support by the Natural Sciences and Engineering Research Council of Canada (NSERC grant numbers RGPIN-2014-05286 and RGPIN-2017-05411; plus an NSERC internship scholarship for CM) and by the Fond de Recherche Nature et Technologie, Quebec.

## Conflict of Interest

The authors declare that the research was conducted in the absence of any commercial or financial relationships that could be construed as a potential conflict of interest.

## Publisher’s Note

All claims expressed in this article are solely those of the authors and do not necessarily represent those of their affiliated organizations, or those of the publisher, the editors and the reviewers. Any product that may be evaluated in this article, or claim that may be made by its manufacturer, is not guaranteed or endorsed by the publisher.

## Footnotes

^1^ https://megasun.bch.umontreal.ca/RNAweasel/

^2^ https://www.ncbi.nlm.nih.gov/genome/browse#!/organelles/fungi

^3^ https://ftp.ncbi.nlm.nih.gov/blast/db/FASTA/nt.gz

^4^ https://github.com/BFL-lab/RNAweasel

^5^ https://megasun.bch.umontreal.ca/cgi-bin/mfannot/mfannotInterface.pl
