# Supplementary material for: Refining Mitochondrial Intron Classification With ERPIN: Identification Based on Conservation of Sequence Plus Secondary Structure Motifs
Source: Front Microbiol. 2022 Mar 18;13:866187. doi: 10.3389/fmicb.2022.866187 (PMC8971849; doi:10.3389/fmicb.2022.866187)
Supplement: Supplementary file 1 [file Table_1.DOCX]

**Supplementary Information**

**Supplementary Table S1**. **NCBI database summary.**

| Database | Total accs.^1^ | Total fungal accs. | Unique fungal recs.^2^ | Fungal recs. missing seqs.^3^ |
| --- | --- | --- | --- | --- |
| Organelle Genome Database | 22679 | 1351 | 896 | 169 |
| Genome Reports | 1509 | 469 | 447 | 169 |
| RefSeq | 11982 | 496 | 496 | 16 |
| Nucleotide | 107445 | 2274 | 2227 | 3 |

^1^ Accessions (accs.) include both RefSeq and GenBank IDs.

^2^ Records (recs.), in GenBank format, contain the sequences and their annotations, if any, which can be referenced by multiple different accessions.

^3^ GenBank records typically contain the genomic sequence but it is not always the case.

**
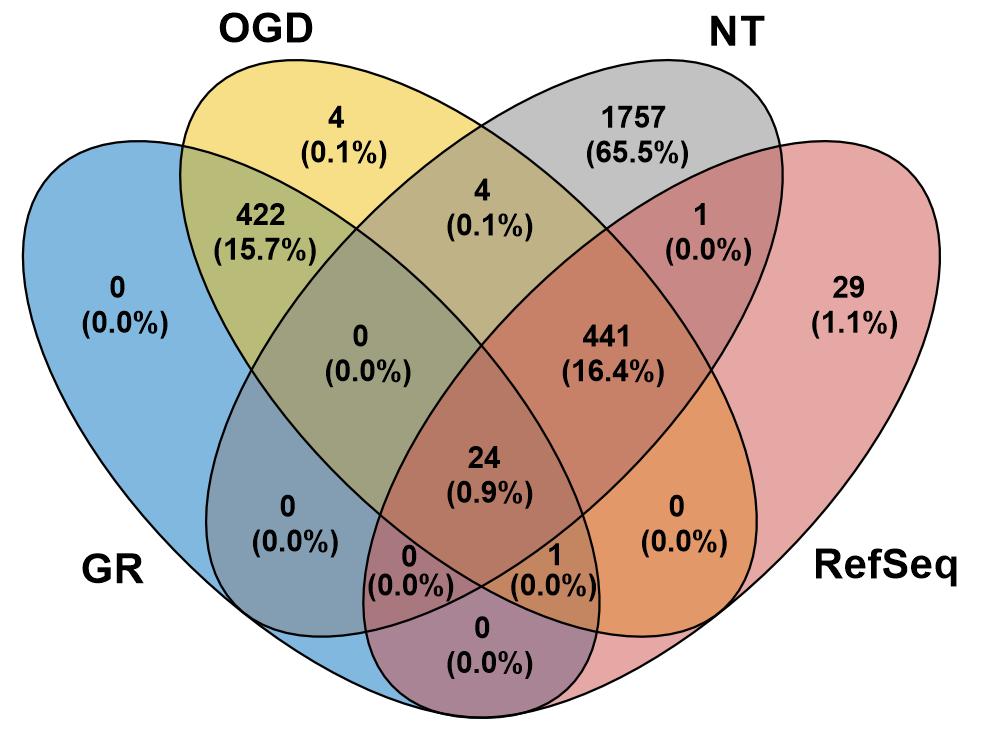
**

**Supplemental Figure S1**: **Overlap in records between NCBI databases.** Overlaps in GenBank records across the Genome Reports (GR), Organelle Genome Database (OGD), RefSeq and Nucleotide (NT) databases. The NT collection contains an order of magnitude more records than the other three.

**
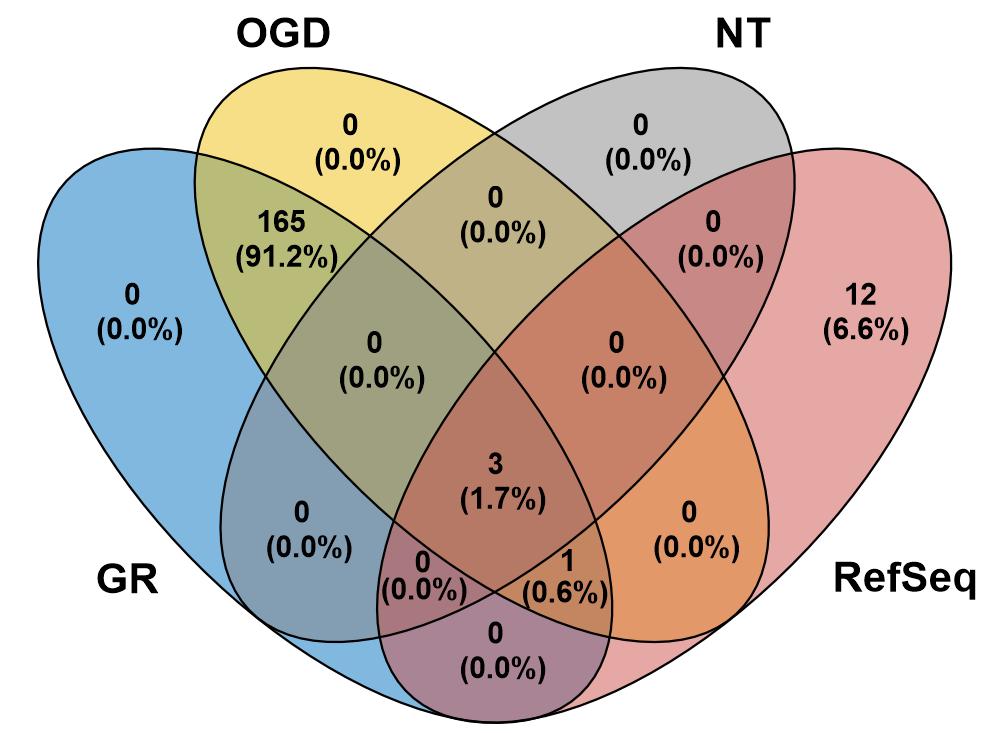
**

**Supplementary Figure S2**. **GenBank records with missing sequences across NCBI databases.**

Overlaps in incomplete GenBank records across the Genome Reports (GR), Organelle Genome Database (OGD), RefSeq and Nucleotide (NT) databases. The NT collection contains an order of magnitude more records than the other three.
